# Sales through note-sharing: influences on the shopping behavior of “Xiaohongshu” users

**DOI:** 10.3389/fpsyg.2024.1334637

**Published:** 2025-05-02

**Authors:** Guanghong Xie, Xiyuan Wang

**Affiliations:** College of Fashion and Design, Donghua University, Shanghai, China

**Keywords:** Sales through note-sharing, “Xiaohongshu”, shopping behavior, interface design, knowledge sharing, social factors

## Abstract

**Introduction:**

To attract more users and promote purchase habits, social e-commerce platforms constantly propose new interaction techniques and marketing strategies. “Xiaohongshu” immediately became famous due to its popular “grass-planting” function. The platform established the “Sales through note-sharing” approach to interrupt the cycle of “planting without uprooting.” The purpose of this study is to investigate the factors influencing online purchase intention of the “Xiaohongshu” Sales through note-sharing model from a human-computer interaction standpoint, as well as the relationships between these factors. To do this, we expanded on the TAM model by including five variables: social identity, social comparison, and knowledge sharing Willingness, interface design, and purchase intention form 12 hypotheses.

**Methods:**

We gathered 287 valid replies from “Xiaohongshu” users and tested them with SPSS and AMOS.

**Results:**

According to the study findings, interface design has a greater impact on purchase intention than knowledge-sharing willingness and behavioral intention to use. Interface design significantly influences knowledge sharing Willingness and social identity significantly influence social comparison, which in turn significantly affects interface design. These results underscore the crucial role of interaction factors, particularly interface design, in purchase intention and the Sales through note-sharing model.

**Discussion:**

This suggests that “Xiaohongshu” can enhance the Sales through note-sharing model by improving interface design to further enhance users’ purchase intention and solidify the “grass-planting and uprooting” loop. In theoretical terms, this study extends the TAM model by integrating social factors (social identity, social comparison, knowledge sharing willingness) and interaction factors (interface design), enriching research in the fields of online purchasing and human-computer interaction on social e-commerce platforms. It also provides relevant insights for stakeholders.

## 1 Introduction

Social e-commerce platforms are developing rapidly, and new technologies and features are the ongoing competitiveness of the platforms to retain and strengthen users’ purchase intention (Chiang et al., 2019; Kuo and Chen, 2020; Sarker et al., 2020; Mafruchah and Hartono, 2023; Liu and Zhang, 2024; Zhang et al., 2024). Many large-scale social e-commerce platforms have launched technology model exploration and achieved some success. For example, INS’s shoppable posts page allows users to conveniently complete their shopping experience in this post (Ertekin, 2017); Facebook’s personalized social ads and promotions significantly influence users’ shopping decisions (Das et al., 2018); and Pinduoduo promotes both social interaction and purchase intention through group activities and link sharing (Yang Q. et al., 2022). According to the study, it was found that China’s online retail sales amounted to 13.79 trillion yuan, an increase of 4% year-on-year. Among them, platforms under the user-generated content (UGC) model in China play an important role in promoting online purchasing behavior (Lyu et al., 2023); for example, the TikTok app, where brands can collaborate with users to allow them to share shopping tips and product recommendations in videos, allows users to make purchases of goods through links to products hung by bloggers, completing the closed loop of commerce (Ramadhani et al., 2023). Xiaohongshu is a popular Chinese app that combines social networking and online purchasing (Liu, 2023). Xiaohongshu is a leader in China’s UGC e-commerce sector, featuring a large amount of user-authentic review content that attracts a large number of young users and facilitates shopping decisions (Liu et al., 2022); according to a research report, “Xiaohongshu” reached 200 million monthly active users in January 2022 (Liu et al., 2022). The process of “grass planting” (Zhe, 2020) is that the head KOL creates momentum, the KOC spreads word-of-mouth feedback, the brand places and promotes in layers, and the brand is publicized through notes. According to the relevant research report, in 2023, the first thing that Xiaohongshu should do is make more efforts on the strong point of “grass planting”. The actual reason behind the “Xiaohongshu” excavation of the “grass-planting economy” is to get rid of the predicament of “only planting grass but not pulling it out” (Wang, 2020); for example, in the past, after learning about product reviews on “Xiaohongshu”, they purchased products through Taobao, Jingdong, and other platforms. In order to complete the “planting and pulling weeds closed loop”, “Xiaohongshu” tried to launch the “Sales through note-sharing (STNS)” mode, which formed the “B2K2C model: connecting brands KOC (Key Opinion Consumers)-consumers” business closed loop. So far, many users have started a second career through this mode and become self-media professionals with considerable profits. This model maximizes the advantages of Xiaohongshu’s “grass planting” and weakens the advertising attribute, which is an innovative attempt.

Although numerous studies have explored users’ purchase intention (PI) and behavior on social e-commerce platforms (Mensah, 2022; Pingping and Ting, 2022; Kamdjoug, 2023), there is still a significant gap in terms of incorporating research under the human-computer interaction perspective (Abdelsalam et al., 2021; Miao and Shu, 2021). Current research focuses mainly on social factors and user behavior, while in-depth studies on the practical applications and potential mechanisms of human-computer interaction on social e-commerce platforms have not yet received sufficient attention. In order to construct a mature research model to understand users’ purchase intentions on social e-commerce platforms, in-depth research is needed to study the influence of social factors and interaction factors on users and platforms on social e-commerce platforms. However, there is relatively limited research on the influence of social factors combined with interaction factors on purchase intention on social e-commerce platforms (Pingping and Ting, 2022). Therefore, existing studies are not necessarily applicable to explain and discuss this particular situation.

Many scholars have conducted research on Xiaohongshu in the past, mostly focusing on the characteristics of generated content, content management, lifestyle, community interaction, etc. (Yan, 2022; Yuan et al., 2022; Jiang and Liu, 2023; Lin and Shen, 2023; Sun and Ly, 2023), but less on the underlying logic of the platform’s new features from the perspective of human-computer interaction (Yang J. et al., 2022; Yang Q. et al., 2022).

Yuan et al. (2022) conducted a study on the purchase intention of Xiaohongshu based on the technology acceptance (TAM) model and social factors but did not take into account the influence of human-computer interaction (HCI) factors, and Yang J. et al. (2022) investigated the influence of live broadcasting e-commerce on impulse purchases from the perspective of HCI but did not incorporate social factors. Lin and Shen (2023) studied the purchase intention of “Xiaohongshu” based on the stimulus organism and response (SOR) theory by incorporating product factors and social factors. What distinguishes this study from other studies is that it takes figurative STNS mode as an example and innovatively integrates interaction factors and social factors based on the Technology Acceptance Model (TAM) when analyzing purchase intention. Unlike past studies that mainly focus on social factors, our approach emphasizes the human-computer interaction perspective as the dominant way of observing the new features on social e-commerce platforms, and unlike studies focusing on the impact of interaction factors on purchase intentions, our approach can play an important role in observing social factors. can contribute to the in-depth observation of social factors, categorizing the three variables of Social Identity Theory (SIT), Social Comparison Theory (SCO), and Knowledge Sharing Willingness (KSW) as social factors, which are better able to observe the influence of an individual’s psychological characteristics and motivation in community activities.

The above-related analysis reflects the diversity and rapid changes in the online shopping space under social e-commerce platforms. These platforms have changed the way consumers shop and have impacted the e-commerce industry (Kansal, 2015; Sarker et al., 2020; Rosário, 2022). Therefore, this paper carries out the following research questions:

RQ1: What are the key factors that influence the shopping behavior of users in “STNS”?

RQ2: To what extent do social and interactive factors influence users’ assessments of the STNS model on the “Xiaohongshu” platform and whether they use it for electronic transactions? What are the potential mechanisms for these effects?

RQ3: Does the TAM model incorporating social and interaction factors change the factors that influence users’ shopping behavior on social e-commerce platforms? What are the specific manifestations of this influence?

The study is divided into the following sections: section 1 is an introduction; Section 2 includes the literature review and research hypotheses; Section 3 is the research method; Section 4 includes the data analysis and results; and Section 5 is the discussion and conclusions.

## 2 Literature review

In today’s digital age, social e-commerce platforms successfully blend social interactions and business transactions (Kumar et al., 2019). For example, platforms such as “Xiaohongshu”, “Zhihu,” and “INS” feature UGC while emphasizing the key role of users in PI. By promoting KSW, SCO, and SIT, these platforms strengthen group identity among community members and individual social identity (Wang and Xu, 2022). On these platforms, user-generated content plays a key role in shaping perceptions, building trust, and influencing purchase intentions (Li and Wang, 2017). User engagement behavior depends heavily on social interactions, technological factors, and motivational factors (Busalim et al., 2021). Social e-commerce platforms use social interactions to achieve business goals and realize the connection between user behavioral intention to use (ITU) and PI. In the field of e-commerce, Taobao and Amazon focus on the interactive experience and personalized recommendation mechanism brought by interface design (ID), and excellent human-computer interaction plays an important role in influencing users’ perceived usefulness (PU) and perceived ease of use (PEU) of the platform, KSW, and PI. “Xiaohongshu” is a popular UGC shopping and sharing community in China that contains content communities and e-commerce modules (Liu et al., 2022). Yan (2022) study found that “Xiaohongshu” combines social interaction and business transactions to provide a unique online shopping experience. The “Xiaohongshu” STNS model is a new feature that allows merchants and celebrities to drive e-commerce transactions through bloggers’ collaborative notes. Note-carrying weakens the “advertising” attribute and strengthens the “grass planting” attribute, which improves the user experience. To better understand the ITU and PI of users on social e-commerce platforms, we will introduce the TAM model and take “Xiaohongshu” as an example to conduct an empirical study.

### 2.1 TAM

TAM was originally proposed by Davis (1989) to explain users’ attitudes toward technology adoption (AIT) and ITU. This model focuses on two key factors, “PU” and “PEU”, which are critical to users’ willingness to adopt new technologies. Based on the TAM model, subsequent studies have extended it by applying it to different domains and emerging technologies, including the UTAUT model, mobile applications, social media, e-commerce, online shopping, the Internet of Things (IoT), and healthcare IT (Ammenwerth, 2019; Harnadi et al., 2022; Sari, 2022). With the rise of social e-commerce platforms, the application of TAM modeling has become particularly important, and the differences in human-computer interaction under different social e-commerce platforms are worth studying and exploring as an emerging technology (Impedovo, 2021; Zhang, 2023). Xu et al. (2022) used the TAM model to study the Taobao live shopping platform. Li and Li (2022) used the TAM model to study the brand communication of Pinduoduo. In the study of Xiaohongshu, Yuan et al. (2022) expanded the TAM model with Key Opinion Leader (KOL) and Trust (TR) variables to study the impact of Xiaohongshu’s user-generated content characteristics on consumers’ purchase intention. The results show that KOL and TR have a significant effect on purchase intention. Zhong (2022) used SCO to study the effect of physical appearance and body on the socialization of female college students in Xiaohongshu. We hypothesize that on the social e-commerce platform Xiaohongshu, users are influenced by social identity, which generates social comparisons, which affect human-computer interaction and ultimately affect the quality of user-generated content and willingness to share, which in turn affects willingness to buy. In past research on social e-commerce platforms, the TAM model was usually used to measure users’ willingness to use and continue to use them (Sari, 2022; Zhu et al., 2022), which mainly focused on the interests of platforms and merchants and less comprehensively discussed the interests of consumers from the perspective of human-computer interaction. This study, in response to previous findings and recommendations, extends the scope of the study to include four external variables, namely SIT, SCO, ID, and KSW, to empirically investigate the effects of these external variables on the PI of Xiaohongshu users.

### 2.2 Perceived ease of use

PEU determines the users’ experience and efficiency in using a new technology, which in turn affects their willingness to accept and adopt the technology (Tsai et al., 2022; Saksono and Untoro, 2023; Wang, 2023). Keni (2020) study shows that PEU is a key factor influencing consumers’ willingness to repurchase, and it is also an important determinant of the PU of a product or service. In addition, related studies have shown that PEU is used to positively assess PU in the field of social e-commerce platforms (Marso, 2022; Priyatma and Eka, 2022; Siagian et al., 2022). In this study, PEU was identified as user experience and utilization efficiency in “Xiaohongshu”. Therefore, we hypothesized:

**H1** PEUs have a positive impact on PUs using Xiaohongshu.

### 2.3 Perceived usefulness

PU determines users’ perceptions and expectations of the actual benefits of a new technology when they use it, which in turn affects whether they see it as valuable to their needs and goals (Ambalov, 2021; Ghani et al., 2022; Park and Lee, 2022). Related studies have shown that PUs have an impact on consumer attitudes toward new technologies on social media and social e-commerce platforms (Purwianti, 2019; Cai, 2022; Siagian et al., 2022). In this study, PU was identified as users’ assessment of the value of the platform’s features in “Xiaohongshu”. Therefore, we hypothesize:

**H2** PU has a positive impact on AIT using the Xiaohongshu.

### 2.4 Attitude

AIT determines the emotions and attitudes of users when using new technologies, including their favorability, willingness to accept the technology and positive or negative perceptions of its use (María-Dolores and Belarmina, 2008). Xu and Ahn’s (2022) study on TikTok confirms that user attitudes toward use play an important role in determining consumers’ feelings and attitudes toward new technologies. In this study, AIT was identified as the user’s attitude toward “Xiaohongshu”. Therefore, we hypothesized:

**H3** AIT has a positive impact on ITUs using the Xiaohongshu.

### 2.5 Behavioral intention to use

ITUs determine the willingness of users to take action (buy, use, recommend, etc.) when using new technologies (Doleck et al., 2018; Mahardika et al., 2019; Sathish and Patankar, 2019). ITU is usually influenced by an individual’s PU, PEU, and AIT factors toward technology (Hess et al., 2014; Cho and Sagynov, 2015; Shropshire et al., 2015). Myra and Hird (2023) found that ITU has a positive impact on the PI of e-commerce platforms when Vietnamese consumers utilize them for online shopping and payment. In this study, ITU was identified as the behaviors that users adopt toward “Xiaohongshu” STNS. Therefore, we hypothesized:

**H4** ITUs have a positive impact on PIs who use the Xiaohongshu.

### 2.6 Social identity theory

SIT is an individual’s perception of his or her position and role in a particular social group (Verkuyten, 2021). Individuals form group identities through social interactions, which are closely related to SIT (Michael and Hogg, 2016). The theory has been widely used in user studies of social media and social e-commerce platforms (Tseng and Kuo, 2015; Jacobsen and Barnes, 2017; Qiao and Wei, 2021; Bazi et al., 2023). In this study, taking “Xiaohongshu” as an example, users cultivate social identity with a specific social group by searching for content, viewing content preferences, interacting with other members, sharing shopping tips, and establishing social relationships; this process helps to form group profiles (group portraits) in the personalized recommendation mechanism of “Xiaohongshu” (Liu et al., 2022; Yu, 2023). Through content sharing and shopping functions, “Xiaohongshu” encourages users to build social relationships and share shopping experiences on the platform, thus enabling social interactions (Lin and Shen, 2023). Therefore, users on Xiaohongshu have the opportunity to build and consolidate their SIT and recognize themselves as part of a social group. The purpose of this study is to explore how the SIT of Xiaohongshu users affects their SCO; therefore, we hypothesize that:

**H5** SIT has a positive impact on the SCO of the Xiaohongshu.

### 2.7 Social comparison theory

SCO means comparing oneself to other members to assess one’s performance in the group (Janelle and Beadle, 2022; Jin, 2022). Research has shown that individuals tend to assess themselves by comparing themselves to others (Garcia et al., 2013). SCO is commonly used in research in the areas of body image management and mental health (Arigo et al., 2021). In addition, SCO has also been applied in research on social media and social commerce platforms (Barrós-Loscertales et al., 2017; DiCosola and Neff, 2020); it is usually used to study the usage behavior and shopping behavior of users within a community. In “Xiaohongshu”, users can view shopping tips, product reviews, and shopping pictures shared by other users for comparison (Zhong, 2022); through comparison, they can then better evaluate their shopping decisions and shopping choices. This hypothesis is proposed because current research on Xiaohongshu has not yet fully explored the integration of social factors and users’ interests from the perspective of human-computer interaction (HCI); this hypothesis can help users gain a deeper understanding of how they participate in the social community of Xiaohongshu and how SCO affects their shopping decisions and shopping choices. Groups and how SCO affects their perceptions of ID; furthermore, it provides insights for Xiaohongshu to iterate on its products and provides a basis for merchants to conduct user research. Therefore, we hypothesized:

**H6** SCO has a positive impact on the ID of the Xiaohongshu.

### 2.8 Interface design

ID is a key component of human-computer interaction that focuses on designing the way users interact with computer systems (Aslan and Aslan, 2022; Sebastian and Nugraha, 2022; Sun, 2022). The ID of a social e-commerce platform is crucial and includes not only visual elements such as layout, colors, and icons but also the design of navigation, search functions, and interactions (Yang J. et al., 2022). Studies have shown that shopping websites with diverse information layouts are more likely to increase consumer satisfaction than complex interface layouts (Xiang et al., 2016; Sohn and Moritz, 2017). In the case of INS, for example, its clean and intuitive design and navigation features enable users to easily browse and purchase social media-related products. Similarly, Amazon provides users with a better experience through its ID and personalized recommendation features, highlighting the critical role of ID for PEU. This suggests that good ID can improve the user experience, reduce cognitive burden, and increase user efficiency, thus promoting better utilization of technology. Therefore, we hypothesize:

**H7** ID has a positive impact on PEUs that use Xiaohongshu.

ID not only affects the attractiveness of the platform but also directly shapes the user’s perception of functionality and shopping experience (Jung, 2017; Fu et al., 2019; Özmen et al., 2022). Hajli (2015) emphasized the importance of user ID on social e-commerce platforms, especially in providing diagnostic and detailed information. Rodríguez Hidalgo et al. (2015) emphasized how social sharing, as a form of social support, enhances the perceived usefulness of users, which is different from traditional vendor advertising. Research has shown that poor ID can lead to users being hindered from utilizing the functionality of an application (Sridevi, 2014). Taking Pinterest as an example, its image-sharing and favorites-based social e-commerce platform has successfully integrated the interface with shopping interactions, enabling users to easily browse and save images of products, which has inspired users to PU the platform and increase PI. TikTok, for example, has a unique ID that organically integrates short-video sharing with commodity recommendations; users may find commodity-related content containing purchase links when browsing short videos, realizing a smooth transition between entertainment and information acquisition and increasing users’ PU to the platform. “Xiaohongshu” combines the attributes of community and e-commerce, introducing the “STNS” model. We predict that this model will be seen as efficient and practical. Therefore, we hypothesize that:

**H8** ID has a positive impact on PUs using the Xiaohongshu.

User ID has a significant effect on knowledge-sharing behavior (Harun and Noor, 2007; Reychav and Wu, 2015). In the field of social e-commerce, the availability and personalized features of IDs significantly affect users’ knowledge-sharing behaviors (Da Costa, 2022). The studies of Jiarui et al. (2022) and Wang and Xie (2022) found that users’ knowledge-sharing behaviors on social e-commerce platforms are influenced by other users’ behaviors (liking, commenting, and sharing), and these social interactions are influenced by platform IDs and PEUs (Arvola, 2006). Users of social media platforms are usually influenced by IDs, and friendly and easy-to-use interfaces can encourage users to be more active in sharing information and engaging in social interactions (Hespanhol et al., 2015). The above phenomenon may also apply to “Xiaohongshu”. Therefore, we hypothesize that:

**H9** ID has a positive impact on KSWs who use Xiaohongshu.

Azam’s (2015) study showed that user ID has a key role in consumers’ ITU and PI on e-commerce platforms. Intuitive and user-friendly IDs enhance the user’s shopping experience, which in turn influences shopping decisions (Cheng, 2019; Wuryandari et al., 2019; Kang et al., 2020). Taking Instagram as an example, its concise and appealing ID and high-quality image and video sharing features successfully integrate social interaction and shopping; users can easily find product tags and links when browsing images and videos, which makes the shopping process more intuitive and promotes consumers’ PI. In summary, excellent HCI mechanisms and IDs have a positive consumer PI impact (Yang J. et al., 2022). The above study can also be applied to the study of “Xiaohongshu”, so we hypothesize that:

**H10** ID has a positive impact on PIs who use Xiaohongshu.

### 2.9 Knowledge Sharing Willingness

Knowledge Sharing Willingness is the willingness of an individual to disseminate organizationally relevant information, ideas, and expertise (Pangil et al., 2020; Razak et al., 2020). Research has shown that an individual’s KSW is influenced by AIT, subjective norms, and organizational climate (Wu et al., 2022). Research has shown that KSW has a positive impact on collaboration, learning, and innovation within organizations and communities (Sensuse et al., 2021; Canestrino et al., 2022). Shateri and Hayat’s (2020) study showed that perceived value significantly affects KSW, and TR mediates between perceived value and KSW. The usefulness of social media tools, especially when users identify with the organization, is an important predictor of KSW (Jin et al., 2019). In virtual communities, knowledge-sharing behaviors are influenced by factors such as TR, perceived relative advantage, and perceived compatibility (Renqiang and Wende, 2022); user-PI and intention to share social business knowledge are significantly influenced by AIT, PEU, and PU (Nandita and Sukaatmadja, 2023); therefore, KSW is an important factor in the final decision of users on social commerce platforms. Users focus on sharing their knowledge with others, which increases their PEU and PU for the platform; in the case of “Xiaohongshu”, users’ KSW may affect their TR for the platform, which in turn affects their assessment of the platform’s PU. Therefore, we hypothesize:

**H11** KSW has a positive impact on PUs using Xiaohongshu.

Kausar et al. (2023), in their study of the Amazon platform, found that reviews are now a major source of information for consumers in e-commerce shopping, providing opinions and feedback about items and significantly influencing shopping decisions. The study showed that when users share their shopping experiences and product reviews on social media, it usually increases their PI (Dieckmann and Unfried, 2020). Users’ knowledge-sharing behaviors on social e-commerce platforms often have an impact on other users, which makes shoppers more likely to purchase products that have been positively evaluated by other users (Zhang and Yu, 2020). In “Xiaohongshu”, users can usually obtain detailed information about products (product reviews, usage tips, beauty tips, etc.) from other users’ knowledge sharing, which helps them make informed purchase decisions (Sun and Ly, 2023). This content not only provides valuable information but also builds the user’s professional reputation in a specific domain (Yang et al., 2016; Lin et al., 2020). For example, when a user shares a detailed review about a new beauty product, other users may follow and trust the user due to their expertise and credibility. This knowledge-sharing willingness not only satisfies the user’s personal interests but also strengthens the user’s position in the social group. This phenomenon further supports the positive association between KSW and PI. Based on relevant literature studies and user behavior analysis, we can hypothesize:

**H12** KSW has a positive impact on PIs who use Xiaohongshu.

**FIGURE 1:**
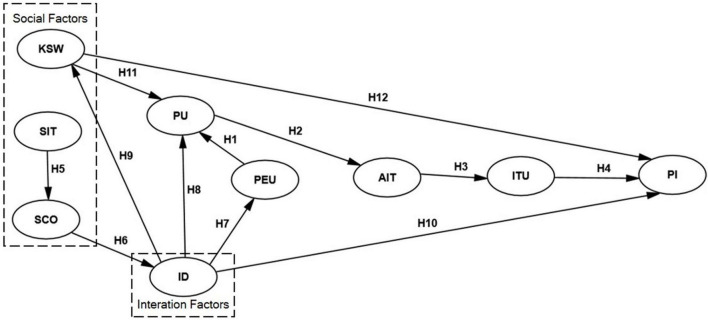
Research model.

All hypothesized associations are shown in Figure 1.

## 3 Methodology

### 3.1 Data collection procedures

A cross-sectional sampling method was used in this study. This study is a web-based survey method conducted in the Chinese context, so no ethical approval was required. In addition, we mentioned informed consent and confidentiality in the introduction of the questionnaire design, which reads, “The results are for academic research use. This research is all conducted anonymously; we will keep it strictly confidential, and it will not affect you. Thank you for your support and cooperation.”. We distributed the questionnaire electronically to ensure that subjects were users of “Xiaohongshu” and had shopped online, excluding individuals who had not used the platform. We distributed the questionnaires to QQ, WeChat and Xiaohongshu. We also utilized the “Mutual Completion Community” in six “Questionnaire Star” accounts to request respondents to complete the questionnaire. In the end, we distributed 330 questionnaires and received 320 responses. After screening, we deleted the data of the sample whose response time was less than 90 seconds and those who chose the same option more than five times consecutively, and finally obtained 287 valid questionnaires, with an effective recovery rate of 90%. Considering that the number of model variable test items developed in this study was 25, Mitchell (2020) suggests that the sample size for SEM should be at least 10–20 times the number of model variable test items; therefore, the sample data for this study should be more than 250 in order to ensure the validity of the study. A total of 287 valid sample data were harvested for this study, which meets the requirements for the conduct of the study and can ensure the validity of the study.

### 3.2 Questionnaire design

The questionnaire consisted of three parts: the first part was an introduction to the purpose, aimed at allaying respondents’ concerns; the second part was a survey of basic information; and the third part was a scale. Scales previously validated in studies related to social media and e-commerce platforms were used, with minor modifications, to achieve the objectives of this study. Related research suggests that new research models should utilize prior scales whenever possible to improve the content and construct validity (Johnston and Finney, 2010). Thus, the scales used to assess PEU (Item count: 3, α = 0.799) and PU (Item count: 3, α = 0.773) were adopted from the study on the role of enhanced flow experience in social media use and its impact on shopping (Hyun et al., 2022). The scale used to assess AIT (Item count: 2, α = 0.802) was adopted from a study of Chinese consumers’ consistent use of mobile ordering apps and a comparative analysis study of users’ acceptance of Facebook and Twitter (Kwon et al., 2014; Wang et al., 2022). The scale used to assess ITU (Item count: 3, α = 0.865) was adopted from a study of e-learning acceptance of knowledge acquisition and knowledge sharing in developing countries (Al-Emran et al., 2021). The scale used to assess SIT (Item count: 2, α = 0.81) was adopted from the study on the role of enhanced mobility experience in social media use and its impact on shopping and the study on the impact of social capital in Chinese virtual communities on social media shopping patterns (Kim et al., 2014; Hyun et al., 2022). The scale used to assess SCO (Item count: 3, α = 0.805) was adopted from the study of factors influencing the purchase intention of face-swapping apps from a social comparison perspective (Ha et al., 2023). The scale used to assess ID (Item count: 3, α = 0.884) was adopted from a study of quality dimensions and satisfaction with a service retailer’s website and a study of factors influencing the acceptance of a generic portal in the Netherlands (van der Heijden, 2003; Kim and Stoel, 2004). The scale used to assess the KSW (Item count: 3, α = 0.874) Knowledge Sharing Willingness scale was adopted from a study of users’ knowledge-sharing willingness in social networks (Zhao et al., 2018) The scale used to assess PI (Item count: 3, α = 0.85) was adopted from the study on the role of social media in changing consumers’ willingness to purchase green products (Nekmahmud et al., 2022). You can find all structural and investigative items in the Supplementary material. The study used a 7-point Likert scale to assess each item (1 = strongly disagree, 7 = strongly agree).

### 3.3 Testing of questionnaires

In the development and revision of the scale, we used both qualitative and quantitative methods. First, we invited five experts to review the questionnaire and refine the items to resolve ambiguities in the items. Then, 50 questionnaires were distributed for the pre-survey. These steps helped to improve validation effectiveness and content validity (Moore and Benbasat, 1991; Gefen, 2002). In addition, our qualitative assessment was supported by an extensive literature review to enhance the qualitative validity of the study.

### 3.4 Data analysis methods

AMOS 27 and SPSS 24 were utilized to measure the quality of the study and investigate the associations between the main variables. Statistical analyses of the data carried out subsequently included (1) demographic characterization. (2) reliability tests (notes: the Cronbach’s coefficient ranges from 0 to 1, and the higher the test result, the higher the reliability (Tavakol and Dennick, 2011). (3) validity analyses (model fit tests (notes: the criteria are detailed in Table 3 (Albright and Park, 2009), convergent and combinatorial reliability tests (notes: according to the standard (Shrestha, 2021), the AVE value should be above 0.5 at least, and the CR value should be above 0.7 at least, to indicate good convergent validity and combined reliability.), discriminant validity tests (notes: the standardized correlation coefficient between dimensions should be less than the square root of the AVE value corresponding to that dimension (Henseler et al., 2015). (4) correlation analyses (notes: the higher the value, the higher the correlation coefficient; the opposite is lower (Cohen et al., 2009). (5) descriptive statistics and normality tests (notes: according to the standard proposed by Finney and DiStefano (2006), the absolute value of the skewness coefficient is within 2, and the absolute value of the kurtosis coefficient is within 7.). (6) structural equations (notes: ***p < 0.001, **0.001 < *p* < 0.01, *0.01 < *p* < 0.05 (Bollen and Noble, 2011).

## 4 Results

### 4.1 Demographic characterization

According to the statistical data of the questionnaire samples in Table 1, the age group of the researched user group mainly focuses on young people aged 18–25, the proportion of respondents with master’s degrees reaches 34.8%, and the proportion of users who have used the “Xiaohongshu” APP for more than 1 year is 64.8%, so it is considered that the questionnaire can be analyzed in the next step of data analysis.

**TABLE 1:** Distribution of sample characteristics.

Variant	Options	Frequency	Percentage
Gender	Male	72	25.10%
	Female	215	74.90%
Age	<18	8	2.80%
	18–25	214	74.60%
	26–35	52	18.10%
	36–45	10	3.50%
	>45	3	1%
Degree	Below Bachelor’s Degree	82	28.60%
	Bachelor’s Degree	93	32.40%
	Master’s Degree (MSC)	100	34.80%
	Ph.D. Degree	12	4.20%
How long	Less than 1 month	31	10.80%
	1–6 months	36	12.50%
	6–12 months	34	11.80%
	1–3 years	120	41.80%
	Over 3 years	66	23%

### 4.2 Reliability test

This study used the scale method to collect data, so the quality of measurement needed to be checked to ensure that the subsequent analysis was meaningful. The internal consistency of the dimensions was first analyzed through Cronbach’s coefficient reliability test. It is generally believed that if the reliability coefficient is below 0.6, the reliability is not credible; between 0.6 and 0.7, the reliability is credible; between 0.7 and 0.8, the reliability is more credible; between 0.8 and 0.9, the reliability is very credible; between 0.9 and 1, the reliability is very credible. Table 2 shows that the values of PU and PEU are between 0.7 and 0.8, which is high confidence; the values of AIT, ITU, ID, SIT, SCO, KSW and PI are between 0.8 and 0.9, which is high confidence; and the summary values are between 0.9 and 1, which is very high confidence.

**TABLE 2:** Reliability analysis.

Variant	Cronbach alpha	Item count
PU	0.773	3
PEU	0.799	3
AIT	0.802	2
ITU	0.865	3
ID	0.884	3
SIT	0.81	2
SCO	0.805	3
KSW	0.874	3
PI	0.85	3
Aggregate	0.9	29

### 4.3 Validity analysis

#### 4.3.1 Model fit tests

According to the results of the model fitness test in Table 3, it can be seen that CMIN/DF = 1.611, which is excellent in the range of 1–3; RMSEA = 0.046, which is excellent; and the values of IFI, TLI, and CFI are 0.962, 0.951, and 0.961, respectively, which are all excellent. Therefore, based on the results of this analysis, it can be concluded that this model has good fitness.

**TABLE 3:** Model fitness test.

Norm	Reference standard	Measurement results
CMIN/DF	Greater than 1 less than 3 is excellent, greater than 3 less than 5 is good	1.611
RMSEA	Less than 0.05 is excellent, and less than 0.08 is good	0.046
IFI	Greater than 0.9 is excellent, and greater than 0.8 is good	0.962
TLI	Greater than 0.9 is excellent, and greater than 0.8 is good	0.951
CFI	Greater than 0.9 is excellent, and greater than 0.8 is good	0.961

#### 4.3.2 Convergent validity and combined reliability tests

On the premise that the CFA model has a good fit, the convergent effect (AVE) and combined reliability (CR) of each dimension in the scale were further examined. The testing process is as follows: first, the standardized factor loadings (ESTIMATE) of each measurement item on the corresponding dimension were calculated by the established CFA model, and then the AVE and CR values were calculated by the formula of AVE and CR. According to the standard, the AVE value should be above 0.5 at least, and the CR value should be above 0.7 at least, in order to indicate good convergent validity and combined reliability.

According to the analysis results in Table 4, it can be seen that in this validity test, the AVE value of each dimension reached more than 0.5 and the CR value reached more than 0.7, which can indicate that each dimension has good convergent validity and combined reliability.

**TABLE 4:** Convergent validity test and combined reliability test for each dimension.

Pathway relationship	Estimate	AVE	CR
PU1 < — PU	0.682	0.533	0.774
PU2 < — PU	0.773		
PU3 < — PU	0.733		
PEU1 < — PEU	0.767	0.571	0.799
PEU2 < — PEU	0.752		
PEU3 < — PEU	0.747		
AIT1 < — AIT	0.851	0.672	0.803
AIT2 < — AIT	0.787		
ITU1 < — ITU	0.827	0.692	0.87
ITU2 < — ITU	0.757		
ITU3 < — ITU	0.905		
ID1 < — ID	0.825	0.722	0.886
ID2 < — ID	0.898		
ID3 < — ID	0.824		
SIT1 < — SIT	0.867	0.688	0.815
SIT2 < — SIT	0.79		
SCO1 < — SCO	0.721	0.585	0.808
SCO2 < — SCO	0.79		
SCO3 < — SCO	0.781		
KSW1 < — KSW	0.843	0.699	0.875
KSW2 < — KSW	0.855		
KSW3 < — KSW	0.81		
PI1 < — PI	0.8	0.657	0.852
PI2 < — PI	0.782		
PI3 < — PI	0.848		

#### 4.3.3 Tests of differential validity

**FIGURE 2:**
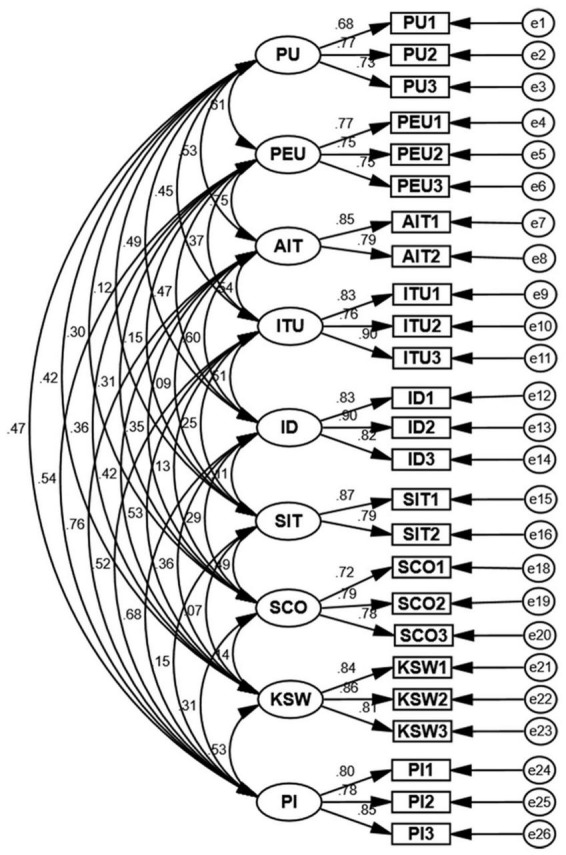
Validation factor analysis CFA model diagram.

According to the analysis results in Figure 2 and Table 5, it can be seen that the standardized correlation coefficients between each dimension in this test of discriminant validity are less than the square root of the AVE value corresponding to that dimension, thus indicating that each dimension has good discriminant validity.

**TABLE 5:** Table of tests of differential validity.

Variant	PU	PEU	AIT	ITU	ID	SIT	SCO	KSW	PI
PU	**0.73**								
PEU	0.611	**0.756**							
AIT	0.528	0.748	**0.82**						
ITU	0.45	0.367	0.542	**0.832**					
ID	0.493	0.465	0.604	0.509	**0.85**				
SIT	0.117	0.154	0.091	0.252	0.109	**0.829**			
SCO	0.3	0.306	0.348	0.128	0.288	0.491	**0.765**		
KSW	0.422	0.358	0.422	0.533	0.361	0.072	0.138	**0.836**	
PI	0.471	0.535	0.757	0.523	0.684	0.146	0.309	0.525	**0.811**

Bolded diagonal font indicates the square root of the AVE.

### 4.4 Descriptive statistics and normality tests

Table 6 shows the normality test results of this study. According to the statistical results of the descriptive analysis, it can be seen that the mean scores of each variable are between 3.97 and 5.48, and the scale scores are 1–7, indicating that this study is above the medium level of awareness of the “Xiaohongshu” social e-commerce platform.

**TABLE 6:** Normality test table.

Variant	Average value	Standard deviation	Skewness	Kurtosis
PU1	5.48	1.17	−0.595	0
PU2	5.41	1.134	−0.583	0.16
PU3	5.47	1.205	−0.706	0.442
PEU1	5.22	1.216	−0.357	−0.536
PEU2	5.21	1.278	−0.522	−0.005
PEU3	4.94	1.363	−0.366	−0.415
AIT1	4.65	1.251	−0.248	−0.179
AIT2	4.67	1.212	−0.154	−0.006
ITU1	4.96	1.281	−0.404	−0.116
ITU2	5.35	1.219	−0.634	0.208
ITU3	5.18	1.286	−0.428	−0.401
ID1	5	1.27	−0.781	0.661
ID2	5.06	1.298	−0.69	0.285
ID3	5.05	1.346	−0.542	0.116
SIT1	5.15	1.363	−0.664	0.135
SIT2	5.15	1.539	−0.544	−0.52
SCO1	5.14	1.182	−0.509	0.124
SCO2	4.82	1.108	−0.002	0.038
SCO3	4.82	1.072	0.041	−0.35
KSW1	4.37	1.648	−0.167	−0.826
KSW2	4.31	1.623	−0.259	−0.635
KSW3	4.74	1.57	−0.559	−0.273
PI1	4.72	1.455	−0.602	−0.016
PI2	3.97	1.553	−0.015	−0.63
PI3	4.36	1.693	−0.369	−0.688

The normality test of each measurement item is carried out using skewness and kurtosis. The data can be regarded as fulfilling the requirement of being close to the approximate normal distribution, and according to the analytical results in Table 6, it can be seen that the absolute values of the skewness and kurtosis coefficients of each measurement question item in the present study are within the standard range.

### 4.5 Correlation analysis

The correlation between each variable was investigated in this analysis using Pearson correlation analysis, and the results of Table 7 show that there is a significant correlation between each variable in this analysis. Among them, PU is most correlated with PEU (*r* = 0.471**), PEU is most correlated with AIT(*r* = 0.471**), ITU is most correlated with KSW(*r* = 0.468**), ID is most correlated with PI (*r* = 0.589**), and SIT is most correlated with SCO (*r* = 0.393**). According to the correlation coefficient results, the correlation coefficient r of each variable is greater than 0. As a result, it is possible to conclude that there is a significant positive correlation between each variable in this analysis.

**TABLE 7:** Pearson correlation analysis between dimensions.

Dimension	PU	PEU	AIT	ITU	ID	SIT	SCO	KSW	PI
PU	1								
PEU	0.471**	1							
AIT	0.435**	0.572**	1						
ITU	0.368**	0.293**	0.460**	1					
ID	0.379**	0.380**	0.529**	0.414**	1				
SIT	0.094	0.123*	0.073	0.198**	0.108	1			
SCO	0.234**	0.260**	0.260**	0.078	0.220**	0.393**	1		
KSW	0.335**	0.285**	0.376**	0.468**	0.314**	0.077	0.106	1	
PI	0.358**	0.412**	0.642**	0.461**	0.589**	0.135*	0.223**	0.430**	1

**Significant at the 0.01 level (two-tailed).

*Significant at the 0.05 level (one-tailed).

### 4.6 Structural equations and results

#### 4.6.1 Model fit tests

Before hypothesis testing, a model fitness test was conducted to test the relationship between the variables in the structural equation model. The results of the test were CMIN/DF = 2.277, RMSEA = 0.067, IFI = 0.912, TLI = 0.899, and CFI = 0.911. According to Table 3, the judgment criteria show that the model fit is good.

#### 4.6.2 Structural equations

**FIGURE 3:**
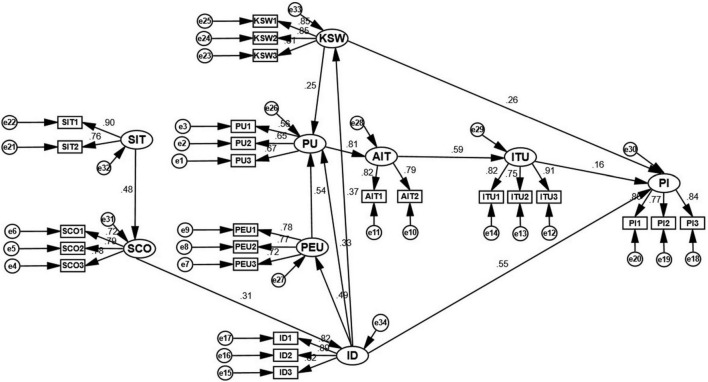
Structural equation modeling path coefficients map.

Figure 3 and Table 8 report the path coefficients of the research model. The results show that hypotheses H1, H2, H3, H5, H6, H7, H8, H9, H10, H11, and H12 are all positive and significant at the 0.001 level; hypothesis H4 is positive and significant at the 0.01 level. The research model showed strong predictive validity and possessed significant explanatory power.

**TABLE 8:** Path analysis study findings.

Hypotheses	Relations	Coefficient	S.E.	C.R.	*P*	Result
H1	PU < — PEU	0.545	0.066	6.735	***	Supported
H2	AIT < — PU	0.808	0.105	9.154	***	Supported
H3	ITU < — AIT	0.595	0.084	8.755	***	Supported
H4	PI < — ITU	0.157	0.066	2.859	**	Supported
H5	SCO < — SIT	0.484	0.053	6.474	***	Supported
H6	ID < — SCO	0.312	0.091	4.495	***	Supported
H7	PEU < — ID	0.489	0.064	6.761	***	Supported
H8	PU < — ID	0.327	0.051	4.635	***	Supported
H9	KSW < — ID	0.374	0.077	5.592	***	Supported
H10	PI < — ID	0.547	0.082	8.399	***	Supported
H11	PU < — KSW	0.248	0.037	4.168	***	Supported
H12	PI < — KSW	0.26	0.064	4.467	***	Supported

***p < 0.001,

**0.001 < *p* < 0.01.

Table 8 shows the path coefficients, S.E., C.R., and P, and the results of the research model. ITU, ID, and KSW have positive and significant effects on the user’s PI (supported H4, H10, and H12). Of these, ID had the most significant effect on users’ PI (β = 0.547). I also had a positive and significant effect on PEU, PU, and KSW (supported by H7, H8, and H9). ID contributed to users’ positive perceptions of the shopping channel, which was reflected in higher levels of PU, PEU, and PI. Similarly, KSW has a positive and significant effect on PU and PI (supported by H11 and H12). KSW contributes to users’ positive perceptions of shopping platform utility and item purchases, which is reflected in higher levels of PU and PI. The research model also shows that PEU has a positive and significant effect on PU (Supported H1). PU has a positive and significant effect on AIT (Supported H2). AIT has a positive and significant effect on ITU (Supported H3). SIT has a positive and significant effect on SCO (Supported H5). SCO has a positive and significant effect on ID (Supported H6). (Hypotheses H10, H4, and H12) Path coefficient analysis shows that ID has a higher effect than ITU and KSW (55% vs. 16% vs. 26%).

## 5 Discussion

The main purpose of this study is to explore the influence of “STNS” on the purchasing behavior of “Xiaohongshu” users. To this end, we conducted a study on online purchase intention based on the TAM model with social and interaction factors. Our study explores the role of the TAM model in the social e-commerce platform “Xiaohongshu” and its positive impact on users’ PI. PEU first affects PU, then PU affects AIT, AIT affects ITU, and ultimately affects PI; in particular, PU has the most significant effect on AIT (β = 0.808). The idea is different from that of previous researchers, and compared with the Yuan et al. (2022) study on the impact of user-generated content characteristics on consumers’ purchase intention, a new research idea is introduced that no longer focuses on the direct impact of PU on PI. First, from the perspective of experimental design, this study focuses more on the impact of the STNS model on users’ interactive behavior and social psychology. Secondly, this study adopts the same theoretical principles as the previous one in that TAM is used, but the difference is that the influence of social factors is explored in greater depth.

First, our study explores the role of SIT in the social e-commerce platform “Xiaohongshu” and its positive impact on users’ SCO. Further, SIT is most correlated with SCO (*r* = 0.393**). Through the analysis of user behavior, this study reveals the multiple values of social factors in the social e-commerce environment. First, social identity plays a key role in building user communities. Users in “Xiaohongshu” resonate with the product reviews and usage tips posted by other users, which in turn drives them to learn more about the product and check out more reviews and usage tips of similar products posted by other users. The study found that this phenomenon further strengthens users’ purchase decisions and knowledge-sharing behaviors. Second, the study also found that there is a positive association between users’ SCO and “Xiaohongshu” ID. In “Xiaohongshu”, the comparison of good and bad information about different products can significantly influence the user’s assessment of the platform’s human-computer interaction, which in turn affects whether the user is willing to purchase and share knowledge with others. This emphasizes the importance of upward and downward comparisons on social e-commerce platforms, especially for merchants that rely on promotional activities. There have been previous studies on social and community factors of Xiaohongshu as variables to study PI (Yuan et al., 2022; Lin and Shen, 2023; Sun and Ly, 2023), where the specific variables are KOL, TR, and community factors (CF). In contrast, this study took three variables as social factors: SIT, SCO, and KSW, and the system of this study is more standardized and comprehensive compared to the former, focusing on the effect of an individual’s psychological characteristics and motivation in the community on PI.

Second, our study explores the role of ID in the social e-commerce platform “Xiaohongshu” and its positive impact on users’ PEU and PU. By analyzing user behaviors and interaction patterns, this study reveals the multiple values of human-computer interaction in the social e-commerce environment. First, user interface design plays a key role in the platform’s daily operation and activity specialization. As a platform where young people are the majority of users, the visual orientation of “Xiaohongshu” influences the activity level of user participation. This visual orientation significantly affects users’ recognition of the community and their sense of self-identity. The study found that this visual orientation further promotes users’ self-perception and social motivation on the platform. Second, the study also found a positive association between Xiaohongshu’s ID and its KSW. On “Xiaohongshu”, good visual effects can significantly influence the knowledge-sharing behavior of other users. This point emphasizes the importance of interactive presentations on social e-commerce platforms, especially for the functional flow of knowledge sharing among users. When platforms and merchants target specific user communities, such as travel enthusiasts, their willingness to share increases through the use of high-quality images and positive experiences. Finally, the study also found a positive association between Xiaohongshu IDs and their PIs. Further, ID is most correlated with PI (*r* = 0.589**). On “Xiaohongshu”, good human-computer interactions can significantly influence the purchase decisions of other users. This emphasizes the importance of visual style on social e-commerce platforms, especially for those with specific community preferences. The results are in line with previous researchers, Yang J. et al. (2022), who also confirmed the influence of ID and on-site atmosphere on consumers’ impulsive purchasing behaviors from the perspective of human-computer interaction.

Finally, our study explores the role of KSW in the social e-commerce platform “Xiaohongshu” and its positive impact on user PU. By analyzing user behaviors and interaction patterns, this study reveals the multiple values of knowledge sharing in the social e-commerce environment. First, knowledge-sharing behavior plays a key role in building and maintaining user community relationships. As a content-driven platform, “Xiaohongshu” has formed a trust-based community network by sharing product reviews and usage tips. This experience-based sharing not only provides practical information for other users but also deepens the relationship between users and promotes community cohesion. It was found that the strengthening of this community relationship further promoted users’ activity and loyalty to the platform. Second, users’ knowledge-sharing behavior is closely related to their status and influence in the community. When users establish an expert image by sharing high-quality content in specific areas, such as beauty, fashion, and knowledge payment, their influence is subsequently enhanced. This not only enhances an individual’s social capital but also brings more user engagement and content generation to the platform. This phenomenon suggests that users’ KSW may be driven by their social identity and self-image construction. Again, the study also found a positive association between users’ KSW and their PI. On platforms such as Xiaohongshu, high-quality UGC can significantly influence the purchase decisions of other users. This emphasizes the importance of user engagement on social e-commerce platforms, especially for brands that rely on user recommendations and reviews to drive sales.

## 6 Conclusion

### 6.1 Theoretical contributions

The theoretical contribution of this study is reflected in the extended application of the TAM model to integrate social factors (SIT, SCO, KSW) and interaction factors (ID) into the TAM model, enriching the research on the application of the TAM model in the field of online purchasing and human-computer interaction under social e-commerce platforms; this extension provides an important theoretical foundation for future research. Specifically, the existing literature mainly focuses on the influence of social factors and information quality on the PI of social e-commerce platforms (Yuan et al., 2022; Lin and Shen, 2023), but less explores the influence of the PI of social e-commerce platforms from the perspective of human-computer interaction. Although Yang J. et al. (2022) studied the impact of ID and live atmosphere on consumer impulse buying behavior under live e-commerce platforms, they did not fully consider the impact of social factors on PI. Second, many studies have focused on the assessment of information sharing and social factors on users’ ITU and PI (Myra and Hird, 2023; Sun and Ly, 2023), whereas the present study is mainly based on interaction factors (ID), and social factors are considered a side factor of influence. Therefore, we delved into the relationship between individuals’ psychological characteristics and motivation in the community, as well as online PI in the context of human-computer interaction in the TAM model. Finally, through the empirical study, we clarified that the effects of ID, ITU, and KSW on PI are all significant. This finding provides solid evidence to further explore the impact of user behavior and PI on social e-commerce platforms. Together, these theoretical contributions emphasize the impact of human-computer interaction on user experience and purchase intention on social e-commerce platforms, providing new perspectives and research directions for studies in related fields.

### 6.2 Practical contributions

The study includes design and marketing strategies for the platform. To begin, the principle of emotionally intelligent interface design can be used to provide a corresponding interface experience to meet the needs of users based on changes in their emotional state; second, the platform can focus on the overall design of the page, including visual elements, layout, and color, to improve the user experience; and finally, the introduction of an honor and reputation system that rewards users for actively participating in the community The implementation of these strategies can refer to the reward programs and promotions of other successful platforms, such as Pinduoduo.

The study provides operational strategies for merchants. First, merchants can learn from the online chat, customer support, and problem-solving provided by WeChat Shop to improve their interactions with customers; second, when posting notes and interacting with customers, they need to pay attention to the visual elements conveyed by words, pictures, symbols, and emojis to satisfy the needs of different groups of users; and finally, merchants can consider launching promotions with partner brands and offering exclusive discount coupons to stimulate group identification and social comparison among users.

The study provides users with strategies for use. First, users should strengthen their control and identification of their own behavior and shopping experience and actively participate in community activities to expand their information sources for smarter and more efficient shopping; second, they should not be frustrated or proud of others’ shopping performance; and lastly, by providing feedback, users can help platforms and merchants better meet their expectations and achieve a win-win situation.

### 6.3 Limitations and future research

The scope and depth of our study are limited in several ways. First, some may argue that HCI can be viewed as a multidimensional construct (e.g., user satisfaction, usability, cognitive load, etc.), whereas in this study it is unidimensional. Secondly, the data collected in this study were mainly focused on China, and the process of collecting the data took only 3 months. Finally, this study uses the TAM model’s expansion of social factors and interaction factors to study consumers’ online purchase intentions.

Therefore, we encourage future studies to examine the multidimensional structural perspective of human-computer interaction. Future studies can expand the sample scope beyond China and moderately lengthen the questionnaire collection period to enhance the external validity and accuracy of the study. Future research can use other models, expand other factors, and add other variables, such as the DOI model, the TPB model, perceived risk, and affective factors, to explain consumers’ online purchase intentions more comprehensively.

## Data availability statement

The original contributions presented in this study are included in the article/Supplementary material, further inquiries can be directed to the corresponding author.

## Author contributions

GX: Writing – review and editing, Writing – original draft, Visualization, Validation, Software, Resources, Project administration, Methodology, Investigation, Funding acquisition, Formal analysis, Data curation, Conceptualization. XW: Writing – review and editing, Supervision, Methodology.

## References

[B1] Busalim Abdelsalam H. Fahad Ghabban Ab Razak Che Hussin (2021). Customer engagement behavior on social commerce platforms: an empirical study. *Technol. Soc*. 10.1016/J.TECHSOC.2020.101437

[B2] Albright J. J. Park H. M. (2009). *Confirmatory factor analysis using AMOS.* LISREL, Mplus, SAS/STAT CALIS. Bloomington.

[B3] Al-Emran M. Mezhuyev V. Kamaludin A. (2021). Is M-learning acceptance influenced by knowledge acquisition and knowledge sharing in developing countries? *Educ. Inform. Technol.* 26 2585–2606.

[B4] Ambalov I. A. (2021). Decomposition of perceived usefulness: a theoretical perspective and empirical test. *Technol. Soc.* 64 0160–791X. 10.1016/J.TECHSOC.2020.101520

[B5] Ammenwerth E. (2019). Technology acceptance models in health informatics: TAM and UTAUT. *Stud Health Technol. Inform.* 263 64–71. 10.3233/SHTI190111 31411153

[B6] Arigo D. Savannah R. Meghan L. Butryn R. (2021). Social comparisons between group members during behavioral weight loss treatment: comparison direction, scale, and associations with weight loss maintenance. *Psychol. Health* 38 429–444. 10.1080/08870446.2021.1967953 34459320 PMC9382642

[B7] Arvola M. (2006). Interaction design patterns for computers in sociable use. *J. Comput. Appl. Technol.* 25 128–139. 10.1504/IJCAT.2006.009063 35009967

[B8] Aslan B. Aslan F. (2022). Examining the user interface development stage in the software development process. *Eur. J. Sci. Technol.* 35 408–416. 10.31590/ejosat.1055996

[B9] Azam A. (2015). The effect of website interface features on e-commerce: an empirical investigation using the use and gratification theory. International Journal of Business Information Systems. 19 205–223. 10.1504/IJBIS.2015.069431 35009967

[B10] Barrós-Loscertales A. Antonio M. Perales J. C. (2017). Social comparisons are associated with poorer and riskier financial decision making, no matter whether encounters are sporadic or repeated. *Spanish J. Psychol.* 19:E57. 10.1017/SJP.2016.55 27647543

[B11] Bazi S. Attar R. W. Adam N. A. Hajli N. (2023). Consumers’ social self- identity drivers on social commerce platforms-based food and beverage. *Br. Food J.* 125 3050–3068 10.1108/bfj-08-2022-0682

[B12] DiCosola F. Neff G. (2020). “Using social comparisons to facilitate healthier choices in online grocery shopping contexts,” in *Extended abstracts of the 2020 CHI conference on human factors in computing systems*, 179–203. 10.1145/3334480.3382877

[B13] Bollen K. A. Noble M. D. (2011). Structural equation models and the quantification of behavior. *Proc. Natl. Acad. Sci.* 108 (Suppl. 3) 15639–15646. 10.1073/pnas.1010661108 21730136 PMC3176611

[B14] Busalim A. H. Ghabban F. Hussin A. R. C. (2021). Customer engagement behavior on social commerce platforms: an empirical study. *Technol. Soc.* 64:101437. 10.1016/J.TECHSOC.2020.101437

[B15] Cai H. (2022). Examining social E-commerce platforms by mediating the effect of perceived usefulness and perceived trust using the technology acceptance model. *J. Organ. End User Comput.* 34 1–20. 10.4018/joeuc.315621

[B16] Canestrino R. Magliocca P. Li Y. (2022). The impact of language diversity on knowledge sharing within international university research teams: evidence from TED project. *Front. Psychol.* 13:879154. 10.3389/fpsyg.2022.879154 35529548 PMC9069179

[B17] Cheng H. (2019). “How does interaction design affect user experience through online shopping interfaces,” in *IOP conference series: Materials science and engineering*, Vol. 573 012076. 10.1088/1757-899X/573/1/012076

[B18] Chiang I.-P. Lin K.-C. Huang C.-H. Yang W.-L. (2019). Influence factors of people purchasing on social commerce sites. *Contemp. Manage. Res.* 15 69–87. 10.7903/CMR.18575

[B19] Cho Y. C. Sagynov E. (2015). Exploring factors that affect usefulness, ease of use, trust, and purchase intention in the online environment. *Int. J. Manag. Inform. Syst. (IJMIS)* 19:21. 10.19030/IJMIS.V19I1.9086

[B20] Cohen I. Huang Y. Chen J. Benesty J. Benesty J. Chen J. (2009). Pearson correlation coefficient. *Noise Reduction Speech Processing* 2 1–4. 10.1007/978-3-642-00296-0_5

[B21] Da Costa T. F. P. N. (2022). “Social media and E-commerce: a study on motivations for sharing content from ecommerce websites,” in *Research anthology on social media advertising and building consumer relationships*, 1681–1702. 10.4018/978-1-6684-6287-4.ch090

[B22] Das S. Mondal S. R. Sahoo K. K. Nayyar A. Musunuru K. (2018). Study on impact of socioeconomic makeup of Facebook users on purchasing behavior. *Rev. Espacios* 39.

[B23] Davis F. D. (1989). Perceived usefulness, perceived ease of use, and user acceptance of information technology. *MIS Q.* 13:319. 10.2307/249008

[B24] Dieckmann A. Unfried M. (2020). Thrilled or upset: what drives people to share and review product experiences? *NIM Market. Intell. Rev.* 12 56–61.

[B25] Doleck T. Bazelais P. Lemay D. J. (2018). The role of behavioral expectation in technology acceptance: a CEGEP case study. *J. Comput. Higher Educ.* 30 407–425. 10.1007/S12528-017-9158-9

[B26] Ertekin S. (2017). “Shoppable videos are in: how do consumers respond,” in *Creating marketing magic and innovative future marketing trends. Developments in marketing science: proceedings of the academy of marketing science*, ed. Stieler M. (Cham: Springer). 10.1007/978-3-319-45596-9_110

[B27] Finney S. J. DiStefano C. (2006). “Non-normal and categorical data in structural equation modeling,” in *Structural equation modeling: a second course*, Vol. 10 eds Hancock G. R. Mueller R. O. (Greenwich, CT: Information Age), 269–314.

[B28] Fu Y. Jiang H. Zhang D. Zhang X. (2019). Comparison of perceptual differences between users and designers in mobile shopping app interface design: implications for evaluation practice. *IEEE Access* 7 23459–23470. 10.1109/ACCESS.2019.2899671

[B29] Garcia S. M. Tor A. Tyrone M. (2013). The psychology of competition: a social comparison perspective. *Soc. Sci. Res. Netw.* 8 634–650. 10.1177/1745691613504114 26173228

[B30] Gefen D. (2002). Reflections on the dimensions of trust and trustworthiness among online consumers. *ACM SIGMIS Database DATABASE Adv. Inform. Syst.* 33 38–53. 10.1145/569905.569910

[B31] Ghani E. K. Ali M. M. Musa M. N. R. Omonov A. A. (2022). The effect of perceived usefulness, reliability, and COVID-19 pandemic on digital banking effectiveness: analysis using technology acceptance model. *Sustainability* 14:11248. 10.3390/su141811248

[B32] Ha Q. A. Le N. Q. T. Nguyen T. T. T. Pham T. T. T. Pham B. A. Le N. Q. L. (2023). How do you wish to appear? An empirical study of factors affecting intention to purchase face-swap apps under social comparison perspective. *Int. J. Hum. Comput. Interact.* 1 1–21.

[B33] Hajli N. (2015). Social commerce constructs and consumer’s intention to buy. *Int. J. Inform. Manag.* 35 183–191. 10.1016/j.ijinfomgt.2014.12.005

[B34] Harnadi B. Prasetya F. H. Widiantoro A. D. (2022). “Understanding behavioral intention to use social media technology: two comparing models, TAM and UTAUT,” in *In 2022 6th international conference on information technology (InCIT)*, (Piscataway, NJ: IEEE), 352–357. 10.1109/InCIT56086.2022.10067645

[B35] Harun A. F. Noor N. L. (2007). “User interface for knowledge sharing using knowledge gardening metaphor,” in *Symposium on human interface and the management of information*, 319–327. 10.1007/978-3-540-73354-6_35

[B36] Henseler J. Ringle C. M. Sarstedt M. (2015). A new criterion for assessing discriminant validity in variance-based structural equation modeling. *J. Acad. Market. Sci.* 43 115–135. 10.1007/s11747-014-0403-8

[B37] Hespanhol L. Tomitsch M. McArthur I. Fredericks J. Schroeter R. Foth M. (2015). Situated interfaces for engaging citizens on the go. *Interactions* 23 40–45. 10.1145/2851200

[B38] Hess T. J. McNab A. L. Basoglu K. S. (2014). Reliability generalization of perceived ease of use, perceived usefulness, and behavioral intentions. *Manag. Inform. Syst. Q.* 38 1–28. 10.25300/MISQ/2014/38.1.01

[B39] Hyun H. Thavisay T. Lee S. H. (2022). Enhancing the role of flow experience in social media usage and its impact on shopping. *J. Retail. Consum. Serv.* 65:102492. 10.1016/j.jretconser.2021.102492

[B40] Impedovo M. A. (2021). “Augmented and emerging transformative interactions with technology: learning in post humanism,” in *Transdisciplinary perspectives on risk management and cyber intelligence*, eds Dall’Acqua L. Gironacci I. M. (Pennsylvania: IGI Global), Vol. 2021 130–144. 10.4018/978-1-7998-4339-9.CH010

[B41] Jacobsen S. Barnes N. G. (2017). On being social: how social identity impacts social commerce for the millennial shopper. *Int. J. Manag. Sci. Bus. Administrat.* 3 38–45 10.18775/IJMSBA.1849-5664-5419.2014.34.1005

[B42] Janelle N. Beadle A. (2022). Investigating the neural bases of social comparison in aging. *Soc. Neurosci.* 17 568–569. 10.1080/17470919.2023.2192959 36942633 PMC10204349

[B43] Jiang Y. Liu Y. H. (2023). Research on the new direction of community marketing - taking the rise of male power of Xiaohongshu as an example. *BCP Bus. Manage*. 36 207–215. 10.54691/bcpbm.v36i.3412

[B44] Jiarui W. Xiaoli Z. Jiafu S. (2022). Interpersonal relationship, knowledge characteristic, and knowledge sharing behavior of online community members: a TAM perspective. *Computat. Intell. Neurosci.* 11. 10.1155/2022/4188480 36262622 PMC9576349

[B45] Jin L. (2022). Social comparison in organizations. *Oxford Res. Encyclopedia Psychol.* 10.1093/acrefore/9780190236557.013.554

[B46] Jin X. L. Zhou Z. Yu X. (2019). Predicting users’ willingness to diffuse healthcare knowledge in social media. *Inform/ Technol. People* 32 0959–3845. 10.1108/ITP-03-2018-0143

[B47] Johnston M. M. Finney S. J. (2010). Measuring basic needs satisfaction: evaluating previous research and conducting new psychometric evaluations of the basic needs satisfaction in general scale. *Contemporary Educat. Psychol.* 35 280–296. 10.1016/j.cedpsych.2010.04.003

[B48] Jung W. (2017). The effect of representational UI design quality of mobile shopping applications on users intention to shop. *Procedia Comput. Sci.* 121 166–169. 10.1016/J.procs.2017.11.023

[B49] Kamdjoug K. J. R. (2023). The influence of social network communication on the buying behavior of Cameroonian consumers on social e-commerce platforms. *J. Enterprise Inform. Manag.* 36 1319–1348. 10.1108/jeim-09-2022-0329

[B50] Kang H. J. Shin J. Ponto K. (2020). How 3D virtual reality stores can shape consumer purchase decisions: the roles of informativeness and playfulness. *J. Interact. Market.* 49 70–85. 10.1016/J.INTMAR.2019.07.002

[B51] Kansal P. (2015). Social commerce and new development in E-commerce technologies. *South Asian J. Market. Manag. Res.* 37 177–178.

[B52] Kausar M. A. Fageeri S. O. Soosaimanickam A. (2023). Sentiment classification based on machine learning approaches in amazon product reviews. *Eng. Technol. Appl. Sci. Res.* 13 10849–10855. 10.48084/etasr.5854

[B53] Keni K. (2020). How perceived usefulness and perceived ease of use affecting intent to repurchase? *Jurnal Manajemen* 24 481–496. 10.24912/JM.V24I3.680

[B54] Kim H. Kim J. Huang R. (2014). Social capital in the Chinese virtual community: impacts on the social shopping model for social media. *Glob. Econ. Rev.* 43 3–24.

[B55] Kim S. Stoel L. (2004). Apparel retailers: website quality dimensions and satisfaction. *J. Retail. Consum. Serv.* 11 109–117.

[B56] Kumar A. Salo J. Li H. (2019). Stages of user engagement on social commerce platforms: analysis with the navigational clickstream data. *Int. J. Electro. Commerce* 23 179–211. 10.1080/10864415.2018.1564550

[B57] Kuo Y.-F. Chen F.-L. (2020). “Social commerce research: a literature review,” in *Proceedings of the the 7th multidisciplinary in international social networks conference and the 3rd international conference on economics, management and technology*, Kaohsiung City. 10.1145/3429395.3429405

[B58] Kwon S. J. Park E. Kim K. J. (2014). What drives successful social networking services? A comparative analysis of user acceptance of Facebook and Twitter. *Soc. Sci. J.* 51 534–544. 10.1016/j.soscij.2014.04.005

[B59] Li Y. M. Li C. (2022). Research on brand communication of social E-commerce pinduoduo. *Acad. J. Bus. Manag.* 4 31–37. 10.25236/ajbm.2022.040205

[B60] Li Y. Wang X. (2017). Social commerce research: definition, research themes and the trends. *Int. J. Inform. Manag.* 37 190–201. 10.1016/j.ijinfomgt.2016.06.006

[B61] Lin B. Shen B. (2023). Study of consumers’ purchase intentions on community E-commerce platform with the SOR model: a case study of China’s “Xiaohongshu” app. *Behav. Sci.* 13:103. 10.3390/bs13020103 PMC9952177 36829332

[B62] Lin X. Xu X. Wang X. (2020). Users’ knowledge sharing on social networking sites. *J. Comp. Inform. Syst.* 62 1–10. 10.1080/08874417.2020.1736690

[B63] Liu J. Zhang M. (2024). Formation mechanism of consumers’ purchase intention in multimedia live platform: a case study of taobao live. *Multimed. Tools Appl.* 83 3657–3680. 10.1007/s11042-023-15666-6 37362664 PMC10193318

[B64] Liu Y. (2023). Analysis of Xiaohongshu’s internet marketing strategy. *BCP Bus. Manag.* 43 110–116. 10.54691/bcpbm.v43i.4629

[B65] Liu Y. Yu Z. Zhang Z. (2022). Analysis of the differentiated competitive strategy of community + E-commerce platform Xiaohongshu. *BCP Bus. Manag.* 24 188–197. 10.54691/bcpbm.v24i.1461

[B66] Lyu W. Wei M. Xian Teo B. S. (2023). Research on the impact of UGC quality on consumers’ purchase intention. *Front. Bus. Econ. Manag.* 7 8–15. 10.54097/fbem.v7i3.5267

[B67] Mafruchah K. N. L. Hartono A. (2023). Antecedents of online purchasing intentions for skintific skincare brand on the social commerce platform. *Asian J. Econ. Bus. Account.* 23 12–28. 10.9734/ajeba/2023/v23i8950

[B68] Mahardika H. Thomas D. Ewing M. T. Japutra A. (2019). Predicting consumers’ trial/adoption of new technology: revisiting the behavioral expectations-behavioral intentions debate. *Int. Rev. Retail Distribut. Consumer Res.* 29 99–117. 10.1080/09593969.2018.1537192

[B69] María-Dolores O.-L. Belarmina B.-D.-V. (2008). Aproximación a las actitudes y percepciones de los usuarios ante las tecnologías de la información. *Profesional De La Informacion* 17 199–204. 10.3145/EPI.2008.MAR.10

[B70] Marso M. (2022). “The effect of perceived ease of use and perceived usefulness on trust, loyalty of E-commerce customers,” in *Proceedings of the 19th international symposium on management (INSYMA 2022)*, Bali, 796–804. 10.2991/978-94-6463-008-4_100

[B71] Mensah I. K. (2022). The factors driving the consumer purchasing intentions in social commerce. *IEEE Access.* 10 132332–132348. 10.1109/access.2022.3230629

[B72] Miao Y. Shu X. (2021). Research on the influence of user perceived overload on information avoidance behavior from the perspective of human-computer interaction. *J. Phys. Conf. Ser.* 1948:012147. 10.1088/1742-6596/1948/1/012147

[B73] Michael A. Hogg A. (2016). *Social identity theory.* Zurich: Springer International Publishing, 3–17. 10.1007/978-3-319-29869-6_1

[B74] Mitchell R. J. (2020). “Path analysis: pollination,” in *Design and analysis of ecological experiments*, (Chapman and Hall/CRC), 211–231.

[B75] Moore G. C. Benbasat I. (1991). Development of an instrument to measure the perceptions of adopting an information technology innovation. *Inform. Syst. Res.* 2 192–222. 10.1287/isre.2.3.192 19642375

[B76] Myra J. Hird A. (2023). Behavioral intention and behavior of using E-commerce platforms for online purchases and payments by Vietnamese consumers. *Conf. Contemp. Econ. Issues Asian Countries* 1 127–156. 10.1007/978-981-19-9669-6_8

[B77] Nandita P. N. Sukaatmadja I. P. G. (2023). Attitude mediates the perceived usefulness and perceived ease of use on continued intention to adopt the halodoc application in denpasar. *Int. J. Econ. Manag. Stud.* 10 42–54. 10.14445/23939125/ijems-v10i1p106

[B78] Nekmahmud M. Naz F. Ramkissoon H. Fekete-Farkas M. (2022). Transforming consumers’ intention to purchase green products: role of social media. *Technol. Forecast. Soc. Change* 185:122067.

[B79] Özmen E. Karaman E. Bayhan N. A. (2022). Users’ emotional experiences in online shopping: effects of design components. *OPUS J. Soc. Res.* 19 6–18. 10.26466/opusjsr.1063894

[B80] Pangil F. Razak N. A. Zin L. M. (2020). Social factors of knowledge sharing willingness: In relational model theory (RMT). *Adv. Bus. Res. Int. J.* 4 9–16. 10.24191/ABRIJ.V4I1.10053

[B81] Park S. B. Lee J. E. (2022). The influence of theory of planned behavior (TPB) on the perceived ease of use and perceived usefulness in accounting information users. *Jeonsan hoe’gye yeon’gu* 20 23–45. 10.32956/kaoca.2022.20.2.23

[B82] Pingping C. Ting Z. (2022). Research on user behavior of social E-commerce. *Acad. J. of Bus. Manag.* 4 51–56. 10.25236/ajbm.2022.040708

[B83] Priyatma J. Eka A. (2022). The impact of perceived usefulness and perceived ease-of-use on repurchase intention of online shopping app users. *Int. J. Soc. Sci. Hum. Res.* 5 4764–4769. 10.47191/ijsshr/v5-i10-49

[B84] Purwianti L. (2019). Peran mediasi perceived usefulness dalam platform c2c e-commerce. *Jurnal Manajemen Pemasaran Jasa* 12 237–252. 10.25105/JMPJ.V12I2.3718

[B85] Qiao S. Wei J. (2021). *Research on the factors affecting customer engagement in social commerce from the perspective of two-factor theory - platform comparison between E-commerce and social media.* 1 8–13. 10.54097/FBEM.V1I3.22

[B86] Ramadhani N. Efni Salam N. Eldapi Yozani R. (2023). Pemanfaatan konten tiktok sebagai media komunikasi pemasaran digital shoppe affiliate pada akun tiktok “indisyindi”. *Pendas J. Ilmiah Pendidikan Dasar* 8 235–261. 10.23969/jp.v8i1.7591

[B87] Razak N. A. Ahmad S. F. S. Rahman Z. A. (2020). Social power and knowledge sharing willingness. *Adv. Bus. Res. Int. J.* 4 35–41. 10.24191/ABRIJ.V4I1.10077

[B88] Renqiang X. Wende Z. (2022). An empirical study on the impact of platform environmental factors on knowledge sharing in virtual communities. *Technol. Soc.* 71 102094. 10.1016/j.techsoc.2022.102094

[B89] Reychav I. Wu D. (2015). The role of user-centered design and usability on knowledge sharing: a school website field study. *Int. J. Knowledge Learn.* 10 16–28. 10.1504/IJKL.2015.071051 35009967

[B90] Rodríguez Hidalgo C. T. Tan E. S. H. Verlegh P. W. J. (2015). The social sharing of emotion (SSE) in online social networks: a case study in Live Journal. *Comput. Hum. Behav.* 52 364–372. 10.1016/j.chb.2015.05.009

[B91] Rosário A. T. (2022). “A look at the new online consumer behavior on social media platforms,” in *Handbook of research on the platform economy and the evolution of E-commerce*, São Leopoldo. 344–369. 10.4018/978-1-7998-7545-1.CH015

[B92] Saksono A. S. Untoro W. (2023). Consumer perceived ease of use and consumer perceived usefulness in using the shopee application in surakarta with discount as a moderation variable. *Eur. J. Bus. Manag. Res.* 8 13–19. 10.24018/ejbmr.2023.8.4.2022

[B93] Sari N. N. (2022). The use of technology acceptance model to explain brand attitude and loyalty intention in E-commerce: the gamification case. *Asean Mark. J.* 14:5. 10.21002/amj.v14i1.1151

[B94] Sarker P. Hughe L. Dwivedi Y. K. Rana N. P. (2020). Social commerce adoption predictors: a review and weight analysis. *Lect. Notes Comput. Sci.* 12066 176–191. 10.1007/978-3-030-44999-5_15

[B95] Sathish S. K. Patankar A. A. (2019). Intent based association modeling for Ecommerce. *Int. Conf. Appl. Nat. Lang. Inform. Syst.* 11608 144–156. 10.1007/978-3-030-23281-8_12

[B96] Sebastian D. Nugraha K. A. (2022). Automatic interface design testing application: case study mobile chat room interface for elderly. *IEE Access* 11–16. 10.1109/IIAI-AAI-Winter58034.2022.00013

[B97] Sensuse D. I. Lestari P. I. Hakim S. A. (2021). Exploring factors influencing knowledge sharing mechanisms and technology to support the collaboration ecosystem: a review. *DESIDOC J. Lib. Inform. Technol..* 41 226–234. 10.14429/DJLIT.41.03.16609

[B98] Shateri K. Hayat A. A. (2020). Investigating the mediating role of organizational trust in the relationship between perceived organizational support and knowledge sharing. *Knowledge Manag. E-Learn. Int. J.* 12 298–314.

[B99] Shrestha N. (2021). Factor analysis as a tool for survey analysis. *Am. J. Appl. Math. Stat.* 9 4–11. 10.12691/ajams-9-1-2

[B100] Shropshire J. Warkentin M. Sharma S. (2015). Personality, attitudes, and intentions. *Comput. Sec.* 49 177–191. 10.1016/J.COSE.2015.01.002

[B101] Siagian H. Tarigan Z. Basana S. Basuki R. (2022). The effect of perceived security, perceived ease of use, and perceived usefulness on consumer behavioral intention through trust in digital payment platform. *Int. J. Data Network Sci.* 6 861–874. 10.5267/j.ijdns.2022.2.010

[B102] Sohn S. Moritz M. (2017). The impact of perceived visual complexity of mobile online shops on user’s satisfaction. *Psychol. Market.* 34 195–214. 10.1002/mar.20983

[B103] Sridevi S. (2014). User interface design. *Int. J. Comput. Sci. Inform. Technol. Res.* 2 415–426.

[B104] Sun Y. (2022). Research on the application method of interaction design in human- machine interface design. *Front. Art Res.* 4 59–62. 10.25236/far.2022.041113

[B105] Sun Y. Ly T. P. (2023). The influence of word-of-web on customers’ purchasing process: the case of Xiaohongshu. *J. China Tourism Res.* 19 221–244.

[B106] Tavakol M. Dennick R. (2011). Making sense of Cronbach’s alpha. *Int. J. Med. Educ.* 2:53. 10.5116/ijme.4dfb.8dfd 28029643 PMC4205511

[B107] Tsai S.-C. Chen C.-H. Shih K.-C. (2022). Exploring transaction security on consumers’ willingness to use mobile payment by using the technology acceptance model. *Appl. Syst. Innovat.* 5:113. 10.3390/asi5060113

[B108] Tseng C. H. Kuo H. C. (2015). What bring to positive behavioral intention of transaction virtual community participants. *Int. J. Inform. Manag. Sci.* 26 68–84. 10.6186/IJIMS.2015.26.1.5

[B109] van der Heijden H. (2003). Factors influencing the usage of websites: the case of a generic portal in the Netherlands. *Inform. Manag.* 40 541–549. 10.1016/s0378-7206(02)00079-4

[B110] Verkuyten M. (2021). *Group identity and ingroup bias: the social identity approach. Human development.* 65 311–324. 10.1159/000519089

[B111] Wang J. Xie J. (2022). Exploring the factors influencing users’ learning and sharing behavior on social media platforms. *Lib. Hi Tech.* 41 0737–8831. 10.1108/lht-01-2022-0033

[B112] Wang S. (2023). Analyze the phenomenon of perceived ease-of-use and perceived usefulness for consumers’ purchase intention. *BCP Bus. Manag.* 39 204–213. 10.54691/bcpbm.v39i.4064

[B113] Wang S. X. (2020). *”Planting grass”/”pulling grass”: a study of fan consumption behavior in the context of social media. Master’s thesis.* Kaifeng: Henan University.

[B114] Wang S. Xu Y. (2022). Complex network-based evolutionary game for knowledge transfer of social e-commerce platform enterprise’s operation team under strategy imitation preferences. *Sustainability* 14:15383. 10.3390/su142215383

[B115] Wang X. Zhang W. Zhang T. Wang Y. Na S. (2022). A study of Chinese consumers’ consistent use of mobile food ordering apps. *Sustainability* 14:12589.

[B116] Wu Y. Hu X. Wei J. Marinova D. (2022). The effects of attitudes toward knowledge sharing, perceived social norms and job autonomy on employees’ knowledge-sharing intentions. *J. Knowledge Manag.* 27 1889–1903. 10.1108/jkm-06-2022-0468

[B117] Wuryandari R. Endah N. Vincentiar P. Permana D. (2019). Buying intention through user interface design. *Eur. Res. Stud. J.* XXII 470–479. 10.35808/ERSJ/1492

[B118] Xiang L. Zheng X. Lee M. K. O. Zhao D. (2016). Exploring consumers’ impulse buying behavior on social commerce platform: the role of parasocial interaction. *Int. J. Inform. Manag.* 36 333–347. 10.1016/j.ijinfomgt.2015.11.002

[B119] Xu C. Li Z. Su J. (2022). Live shopping interactivity, social presence and sustainable consumer purchase intention: based on TAM model. *Int. J. Sustain. Dev. Plann.* 17 2631–2639. 10.18280/ijsdp.170832

[B120] Xu Y. L. Ahn H. K. (2022). Effects of the technology acceptance model and negative perceptions of advertising on TikTok in-feed advertising attitudes: exploring the mediating effect of user flow. *Sahoe gwahag nonchong - gye’myeong daehag’gyo* 41 59–94. 10.18284/jss.2022.12.41.3.59

[B121] Yan Z. (2022). Research on business model of social E-commerce —take Xiaohongshu as an example. *BCP Bus. Manag.* 34 184–193. 10.54691/bcpbm.v34i.3012

[B122] Yang J. Cao C. Ye C. Shi Y. (2022). Effects of interface design and live atmosphere on consumers’ impulse-buying behaviour from the perspective of human- computer interaction. *Sustainability* 14:7110. 10.3390/su14127110

[B123] Yang J. Sia C. L. Liu L. Chen H. (2016). Sellers versus buyers: differences in user information sharing on social commerce sites. *Inform. Technol. People* 29 444–470. 10.1108/ITP-01-2015-0002

[B124] Yang Q. Zhao S. Li J. Lan C. Liu S. Huang W. (2022). The call of social capital in pinduoduo withdrawal activities from the perspective of social network theory. *Front. Bus. Econ. Manag.* 6:46–50. 10.54097/fbem.v6i2.2772

[B125] Yu S. (2023). “Investigation of users experience of social medias personalized recommendation the case of Xiaohongshu,” in *Lecture notes in education psychology and lecture notes in education psychology and public media.* 10.54254/2753-7048/3/2022456

[B126] Yuan Y. Yao J. Li Y. Ying K. Chen J. (2022). Influence of user-generated content characteristics on consumers’ purchase intention taking “Xiaohongshu” as an example. *J. Nonlinear Convex Anal.* 23 2209–2226.

[B127] Zhang Q. Wang Y. Ariffin S. K. (2024). Consumers purchase intention in live-streaming e-commerce: a consumption value perspective and the role of streamer popularity. *PLoS One* 19:e0296339. 10.1371/journal.pone.0296339 38358985 PMC10868799

[B128] Zhang X. Yu X. (2020). The impact of perceived risk on consumers’ cross-platform buying behavior. *Front. Psychol.* 11:592246. 10.3389/fpsyg.2020.592246 PMC7673429 33250830

[B129] Zhang Y. (2023). Study on customer loyalty of cross-border import E-commerce platform based on human-computer interactive user experience. *High. Bus. Econ. Manag.* 10 1–10. 10.54097/hbem.v10i.7907

[B130] Zhao J. Zhu C. Peng Z. Xu X. Liu Y. (2018). User willingness toward knowledge sharing in social networks. *Sustainability* 10:4680. 10.3390/su10124680

[B131] Zhe L. (2020). Analyzing the communication theory behind online “weeding”-taking Xiaohongshu APP as an example. *J. Journalism Res.* 8 51–52.

[B132] Zhong Y. (2022). The influence of social media on body image disturbance induced by appearance anxiety in female college students. *Psychiatr. Danub.* 34 (Suppl. 2):638.

[B133] Zhu Y. Wei Y. Zhou Z. Jiang H. (2022). Consumers’ continuous use intention of O2O E-Commerce platform on community: a value co-creation perspective. *Sustainability* 14:1666. 10.3390/su14031666

