# Supplementary material for: Sales through note-sharing: influences on the shopping behavior of “Xiaohongshu” users
Source: Front Psychol. 2025 May 2;15:1334637. doi: 10.3389/fpsyg.2024.1334637 (PMC12083460; doi:10.3389/fpsyg.2024.1334637)
Supplement: Supplementary file 1 [file Table_1.DOCX]

Supplementary Material

# Supplementary Table

**Supplementary Date 1.** Survey Questionnai.

| Variable | Measurement items | Modified measurement items |
| --- | --- | --- |
| Perceived usefulness  （Hyun et al.，2022） | 1. Using the SNS enables me acquire more information or meet more people. | 1. Using "Xiaohongshu" enables me to acquire more information and connect with a wider range of individuals interested in shopping. |
|  | 2. Using the SNS would improve my efficiency in sharing information and connecting with others. | 2. Utilizing "Xiaohongshu" can enhance my efficiency in sharing information and connecting with others for shopping purposes. |
|  | 3. The SNS is a useful service for communication. | 3. "Xiaohongshu" serves as a valuable communication service for fulfilling shopping needs. |
| Perceived ease to use  （Hyun et al.，2022） | 1. Learning to use the SNS is easy for me. | 1. Learning to use "Xiaohongshu" for my shopping needs was straightforward for me. |
|  | 2. The process of using the SNS is clear and understandable. | 2. The shopping process when using "Xiaohongshu" is clear and easy to understand. |
|  | 3. I find the SNS easy to use. | 3. I find "Xiaohongshu" easy to use for shopping. |
| Attitude  （Wang et al.，2022；Kwon et al.，2014） | 1.Purchasing food through mobile food order apps services is wise. | 1. Purchasing goods through the "Xiaohongshu" service is a wise decision. |
|  | 2.I think Facebook (or Twitter) is helpful to our society. | 2. I believe "Xiaohongshu" is beneficial to our society. |
| Behavioral intention to use（Emran et al.，2021） | 1.I intend to increase my use of the M-learning system. | 1. I intend to enhance my usage of "Xiaohongshu". |
|  | 2. It is worth to recommend the M-learning system for other students. | 2. Suggesting "Xiaohongshu" to others is worthwhile. |
|  | 3. It is worth to recommend the M-learning system for other students. | 3. I'm interested in using "Xiaohongshu" more frequently in the future. |
| Interface Design  （Kim et al.，2021；Heijden，2003） | 1.The website is visually pleasing. | 1. The visual presentation of "note-taking" socialization is aesthetically pleasing. |
|  | 2. The lay-out of the site is attractive. | 2. The layout of "note-taking" socialization is visually appealing. |
|  | 3.The colours that are used on the site are attractive. | 3. The use of colors in the "note-taking" social approach is visually appealing. |
| Social Identity  (Hyun et al.，2022；Kim et al.，2014) | 1. As a member of the community, I am the type of person who likes to engage in my community | 1. As a member of the "Xiaohongshu" app, I am someone who enjoys engaging in activities. |
|  | 2. Iam proud to be a member | 2. I take pride in being a user of "Xiaohongshu." |
| Social Comparison theory  （Ha et al. ，2023） | 1. When I think about others who are doing better than I am, it is threatening to notice that I am doing not so well. | 1. When I come across individuals on "Xiaohongshu" who are performing better than me, it makes me aware of areas where I might not be doing as well. |
|  | 2.When I see others who are doing worse, I experience fear that my situation will decline. | 2.When I see that others' situations are worse on "Xiaohongshu," I worry that my own circumstances might deteriorate. |
